# Acute effects of intravitreal aflibercept injections on intraocular pressure in vitrectomized and silicone-oil-filled eyes: a prospective cohort study

**DOI:** 10.1038/s41598-026-38455-1

**Published:** 2026-02-16

**Authors:** Áron Szabó, Géza Thury, Nóra Baranyi, Ferenc Rárosi, Dóra J. Szabó, Arnold Gale, Edit Tóth-Molnár, Attila Kovács

**Affiliations:** 1 Department of Ophthalmology, University of Szeged, Korányi Fasor 10–11, Szeged, 6720 Hungary; 2 Department of Biostatistics, University of Szeged, Szeged, Hungary

**Keywords:** Aflibercept, Injection, Intraocular pressure, Intra-silicone oil, Vitrectomized, Diseases, Medical research

## Abstract

This prospective, single-center, single-blind, interventional cohort study compares acute intraocular pressure (IOP) changes following intravitreal aflibercept injections in eyes with different vitreous states. 67 eyes of 58 patients, including vitrectomized eyes with (*n* = 22) and without silicone oil tamponade (*n* = 22) and nonvitrectomized eyes (*n* = 23) were analyzed. Intravitreal aflibercept was administered according to guidelines. IOP was evaluated before and immediately after intravitreal injection (IVI) and at 5, 15, 30, 60, and 180 min, day 1, and week 1 postinjection. IOP measurements were taken with iCare-100 rebound tonometry (RBT) and Goldmann applanation tonometry (GAT). The mean participant age was 62.13 ± 14.06 years (male-to-female ratio, 1.48:1). Postinjection IOP increased to the same levels. IOP curve resolution was similar in all vitreous groups, showing no significant difference at any timepoints. Emulsified silicone oil did not affect the IOP curve. Prior IVI count had some influence on the dynamics of IOP spike resolution but did not affect the initial IOP rise. RBT showed moderate-to-good agreement with GAT, except for extremely elevated IOPs where it appeared less consistent. Intra-silicone and post-vitrectomy administration of aflibercept is safe. RBT may be considered for nonglaucomatous cases as an easy alternative to follow-up postinjection acute IOP changes.

## Introduction

Intravitreal injections (IVI) have become the most frequently performed specialized care interventions^1^. The rise in acute intraocular pressure (IOP) following IVIs has been thoroughly discussed in nonvitrectomized eyes^2,3^. The effect of silicone oil (SiO) microdroplet contamination from syringes is also well described; however, whether there is an association with chronic IOP elevation remains controversial^4,5^. The acute and chronic pressure changes following IVI in vitrectomized eyes with or without SiO tamponade have been less investigated. Intravitreal triamcinolone-acetonide (TCA) dose-dependently increases IOP in vitrectomized eyes for approximately 6 months and is postulated to exhibit different changes from nonvitrectomized eyes^6^. Steroids such as TCA are nevertheless associated with a higher incidence of IOP-related adverse events than monoclonal antibody or recombinant fusion protein type anti-vascular endothelial growth factor (anti-VEGF) drugs^7^. Intra-silicone bevacizumab administration has not been shown to increase IOP in a vitrectomized SiO tamponade animal model^8^. Similarly, limited human data are available from individual case reports^9^ and pilot studies^10^. Even the recently published evidence-based guidelines on IVIs in SiO-filled eyes marginally discuss IOP changes due to limited data and a myriad of confounding factors^11^. The administration of intra-silicone anti-VEGF therapy appears to be effective in reducing the macular thickness in diabetic macular edema^12^. As the number of IVIs^13^ and vitrectomy are expected to increase^14^, the detailed dynamics of postinjection IOP in different vitreous cavity states must be elucidated.

This prospective controlled trial aimed to investigate the acute effects of intravitreal aflibercept injections (IVA) on IOP in eyes that have undergone vitrectomy with or without SiO tamponade. To our knowledge, no similar research has been published in the literature so far.

## Methods

### Patient selection

A prospective, single-center, single-blind interventional cohort study was conducted from March 2022 to February 2025. After obtaining informed consent, patients requiring IVA were enrolled in one of the three study arms and analyzed. The G-SiO group included previously vitrectomized eyes that had undergone SiO tamponade (Oxane 1300, Bausch & Lomb, Bridgewater, NJ, USA). The G-PPV group consisted of previously vitrectomized eyes with 23G pars plana vitrectomy (PPV) surgery performed more than 3 months before enrollment, did not have SiO tamponade, or had undergone prior SiO tamponade. The G-NVIT group included eyes with intact vitreous bodies. Being treatment-naïve to IVIs was not a prerequisite for participating in the trial, whereas all participants’ eyes were treatment-naïve to antiglaucoma medications (not even temporary IOP lowering treatment was administered following SiO implantation or PPV surgery) and showed no signs of glaucomatous optic nerve disease.

If both eyes were eligible, both were enrolled in the study, as the treatment method did not differ among the cohorts. Because the IOP investigators were unaware of the cohorts and no rescue treatment was required during the study, the inclusion or exclusion of the second eye was not influenced by the type of bias described in the Guidelines on Design and Reporting of Glaucoma Surgical Trials^15^.

The Ethics Committee of the University of Szeged reviewed and approved the trial (Protocol no. NH_SiOP-001, Reference no. 168/2022-SZTE RKEB). The study was conducted in accordance with the tenets of the Declaration of Helsinki.

This clinical trial is registered and trial protocol is available in the HMA-EMA Real-World Data (RWD) Catalogues. EU PAS number: EUPAS1000000811. Date of registration: 04/11/2025, First published: 06/11/2025.

### Diagnostic procedures

All eyes were evaluated thoroughly. The best-corrected visual acuity was recorded on a standardized early treatment diabetic retinopathy study chart. The axial length (AL), anterior chamber depth (ACD), and central corneal thickness (CCT) were captured with an ARGOS^®^ Swept Source OCT Biometer (Movu - Santec Corporation, Santa Clara, CA, USA). The lens status was recorded. Fundus examination and Heidelberg Spectralis optical coherence tomography (OCT) (Heidelberg Engineering GmbH, Heidelberg, Germany) were also performed to confirm the referring diagnosis and indication for IVA. Undilated dark-room gonioscopy was performed after induction of topical anesthesia with a Magnaview gonio laser lens (Ocular Instruments, Bellevue, WA, USA). Angle structures were identified with the modified Shaffer system. An integrated index (Shaffer360 [S360]) was created for each eye based on the values corresponding to the four quadrants with 16 (4 + 4 + 4 + 4) being the absolute open end of the spectrum with the ciliary body visible in 360° and 0 (0 + 0 + 0 + 0) being the completely closed end with no Schwalbe’s line visible in any of the quadrants. The extent of the iridotrabecular contact (ITC) and the presence of emulsified SiO were recorded in degrees of the arc where applicable. Angles were classified as open or closed based on ITC exceeding 180°^16^. Angle or iris neovascularization as well as gonioscopic angle closure were criteria for exclusion.

IOP measurements were taken with iCare-100 (Icare Finland Oy, Vantaa, Finland) rebound tonometry (RBT) and Goldmann (Haag-Streit, Köniz, Switzerland) applanation tonometry (GAT) in the seated position. An investigator masked to the IOP values performed the measurements, while a separate IOP reader recorded the results. Both were blinded to patients’ vitreous status. The final average of six quick consecutive measurements taken in the manual mode was used for RBT, whereas a single reading was obtained for GAT in all study eyes after topical anesthesia. The concept of repeated GAT readings (average of two readings followed by a third if the difference is > 2 mmHg)^15,17^ was considered during the initial study design but was eventually waived because of the expected steep drop in IOP and resulting confounding effect in the early phase of measurements. The IOP data presented in the study were derived from GAT, whereas RBT measurements were used to establish the level of agreement between the readings. This method was uniformly applied for all time points. IOP was evaluated at baseline (BL) and immediately (within 1 min) following IVA (T1) and 5, 15, 30, 60, and 180 min (T5-180) post-IVA, as well as on day 1 (D1) and week 1 (W1). IOP readings at follow-up visits were recorded with dilated pupils to maintain consistency.

### Study intervention

None of the eyes received IOP-lowering pretreatment immediately before or after injection. The indications for IVA are presented in Table 1.

Every single IVA treatment was administered according to the European Society of Retina Specialists guidelines^18^. In particular, 0.05 mL of 2 mg aflibercept (Eylea, Bayer AG, Leverkusen, Germany) was administered into the vitreous cavity by the same experienced investigator using a 1 mL BD Luer-Lok Tip syringe (Becton, Dickinson and Company, Franklin Lakes, NJ, USA) via a 30-G Medoject hypodermic needle (CHIRANA T.Injecta, Stara Turá, Slovak Republic). The needle entered the sclera using a tunneled technique at an approximate angle of 30°, lifted to 90°, and penetrated the sclera, while directing the injection toward the center of the globe. The needle and syringe were subsequently withdrawn at the same 30° angle to minimize reflux^19^. A sterile cotton swab was applied for 10 s to further prevent reflux when withdrawing the needle^2^.

Immediate appearance of any fluid (except blood) at the injection site following the withdrawal of the needle was classified as reflux and dimensions (perpendicular diameters) measured with calipers in millimeters. All eyes with reflux were excluded from the statistical analysis but were followed according to the study protocol.

Rescue treatment (paracentesis and/or intravenous acetazolamide) was warranted if the patient reported no light perception (NLP) at T1 or if the IOP remained high (> 50 mmHg) at T30.

### Statistical analysis

Sample size calculation was performed using GPower (version 3.1.9.7) for a two-way repeated-measures ANOVA assessing the cohort × time interaction in IOP following IVI. A standardized effect size of f = 0.25 was assumed, corresponding to a medium effect according to Cohen’s conventions.

The analysis was based on three independent cohorts and nine repeated IOP measurements, as dictated by the study protocol. Statistical power was set at 95% to reduce the risk of type II error in detecting clinically relevant differences in IOP response over time. The significance level was set at the standard level α = 0.05. The correlation among repeated IOP measurements was conservatively specified as 0.40, reflecting moderate within-eye correlation. To account for potential violations of the sphericity assumption, a Greenhouse–Geisser correction of ε = 0.50 was applied. Based on these assumptions, the minimum required total sample size was 51 eyes.

Continuous data were expressed as mean ± standard deviation (SD) or median (first quartile [Q1] and third quartile [Q3]) for symmetrical or skewed distributions, respectively. Categorical data were expressed as the number of cases (frequencies) or percentages (relative frequencies).

The relationships between the categorical variables were investigated by the Chi-squared test for independence.

Continuous variables in the three cohorts were compared with the one-way analysis of variance (ANOVA), Welch ANOVA, or Kruskal–Wallis test for symmetrical or skewed distributions, respectively. Post hoc comparisons for Welch ANOVA were conducted by Games–Howell tests.

IOP was analyzed with a linear mixed-effects model including fixed effects for group (vitreous status), time, and their interaction. Patient clustering was modeled with a random intercept, and within-eye repeated measurements. Estimated marginal means for group (vitreous status) at different timepoints were obtained and Bonferroni-adjusted between-group comparisons at each time point were performed. This specification accommodates unequal variances over time, serial correlation within eyes, and patient-level clustering.

IOP levels at all timepoints with respect to all examined parameters such as age, sex, AL, ACD, CCT, lens, and angle status were analyzed also with a linear mixed-effects model.

The agreement between GAT and RBT was analyzed with the Bland–Altman method, and 95% agreement limits were calculated.

Calculations were conducted using IBM SPSS Statistics version 29.0.0.0 (241). P-values < 0.05 were regarded as significant.

## Results

A total of 70 eyes were initially enrolled in the study; however, three eyes were excluded because of reflux, resulting in the analysis of 67 eyes of 58 patients distributed across three cohorts: 22 eyes in the G-SIO group, 22 in the G-PPV group, and 23 in the G-NVIT group. Nine patients (18 eyes) participated in a consecutive bilateral enrollment; four in G-SIO, four in G-PPV, and one in G-NVIT.

The mean age of the study participants was 62.13 ± 14.07 years. The male-to-female ratio was 1.48:1. All patients were of Caucasian ancestry. The BL characteristics of each study arm are presented in Table 2. Patients enrolled in the G-SiO group were significantly younger (*p* = 0.017) and had significantly more treatment-naïve eyes to prior IVIs before administration of the investigational IVA (*p* < 0.001) than those in the other two groups.

The time between SiO implantation and enrollment followed a skewed distribution with a median of 118 days with 84.5 in Q1 and 211.5 in Q3.

None of the eyes required immediate rescue treatment, and no patient reported NLP following IVA at any time point.

**Fig. 1:**
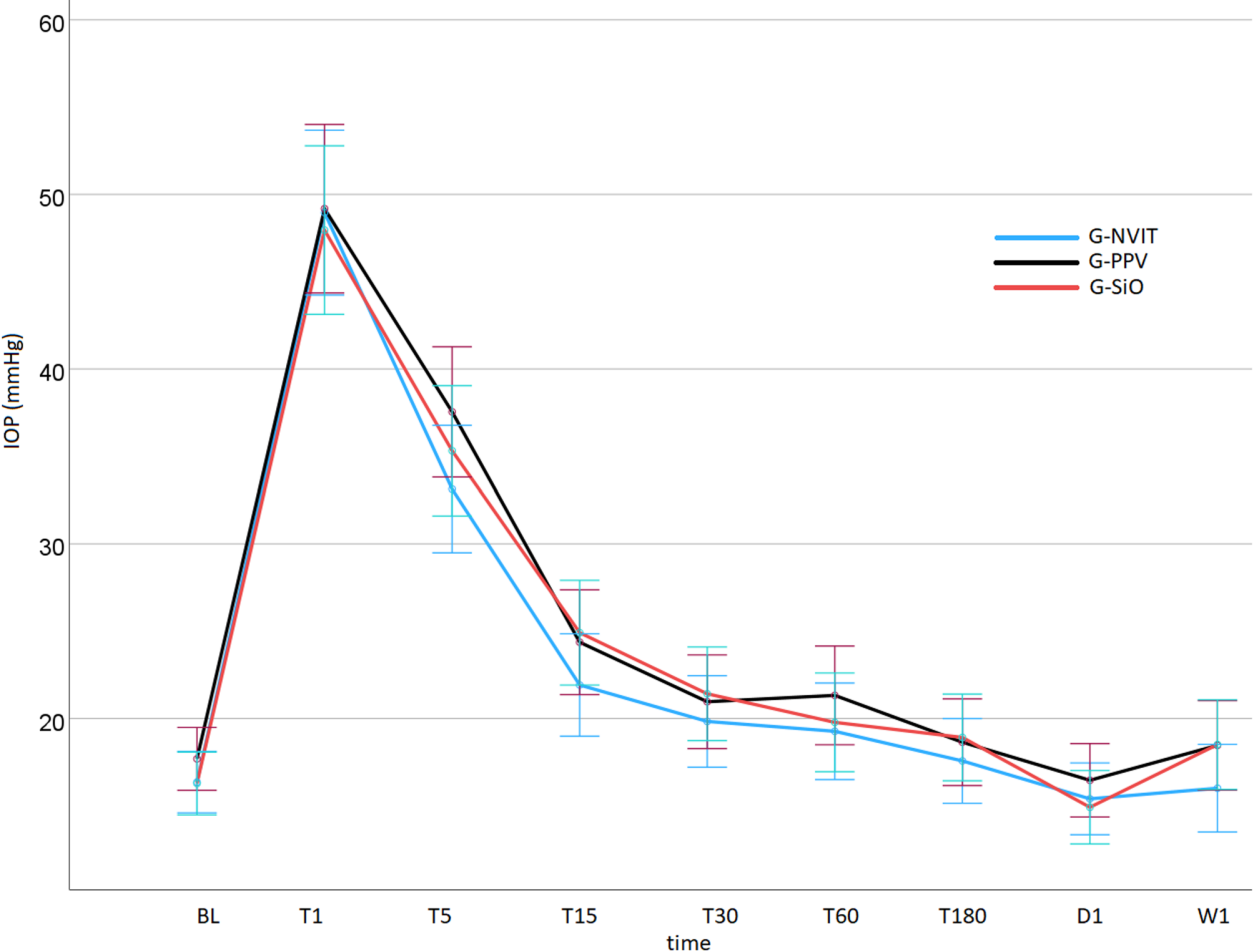
Intraocular pressure resolution curves of the three study arms. (G-NVIT, non-vitrectomized study arm; G-PPV, pars plana vitrectomy study arm; G-SiO, silicone oil study arm). x-axis: BL, baseline; T1, within 1 min post-injection; T5-T180, 5, 15, 30, 60, and 180 min post-injection; D1, day 1; W1, week 1.

A total of 603 GAT-derived IOP data points were analyzed. Post-IVA IOP increased at T1 to 48.95 ± 12.23, 49.18 ± 11.10, and 47.95 ± 10.57 mmHg in the G-NVIT, G-PPV, and G-SiO vitreous groups, respectively, with no significant difference between the groups, and returned to BL levels by T180 regardless of the vitreous status (Fig. 1).

No significant relationship was found between the level or duration of IOP rise and age, sex, AL, ACD, CCT, or gonioscopic angle status (S360 or extent of ITC).

Eyes in G-SiO that had visible emulsified SiO (*n* = 9) covering the trabecular meshwork (45°–180°) and eyes that had no SiO droplets nor emulsified SiO visible in the chamber angle (*n* = 13) did not differ regarding the level of or speed of the resolution of the post-IVA IOP spike.

**Fig. 2:**
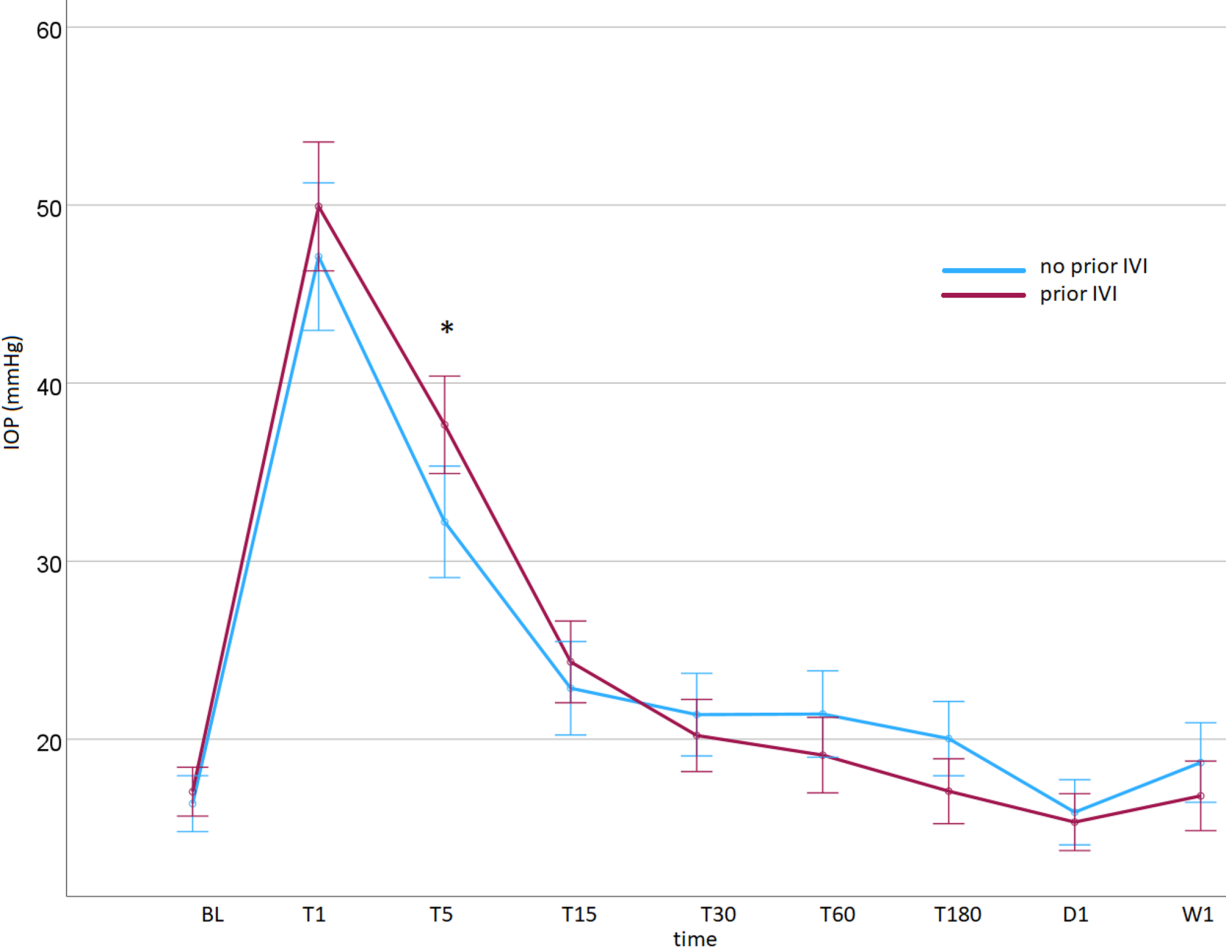
Intraocular pressure resolution curves of eyes that had received prior intravitreal injection (IVI) treatment and eyes that were naïve to IVI treatment. (* - *p* < 0.05). x-axis: BL, baseline; T1, within 1 min post-injection; T5-T180, 5, 15, 30, 60, and 180 min post-injection; D1, day 1; W1, week 1.

Further analysis of the entire cohort with a linear mixed-effects model revealed that the IOP curve resolution did not significantly differ between eyes that had received prior IVI treatment (*n* = 38) and eyes that were naïve to it (*n* = 29) (*p* = 0.121). IOP at BL (*p* = 0.241) and T1 (*p* = 0.441) was not significantly different; however, eyes that had received multiple prior IVIs showed significantly higher IOPs at T5 (*p* = 0.042). No significant difference was observed at later timepoints (Fig. 2).

**Fig. 3:**
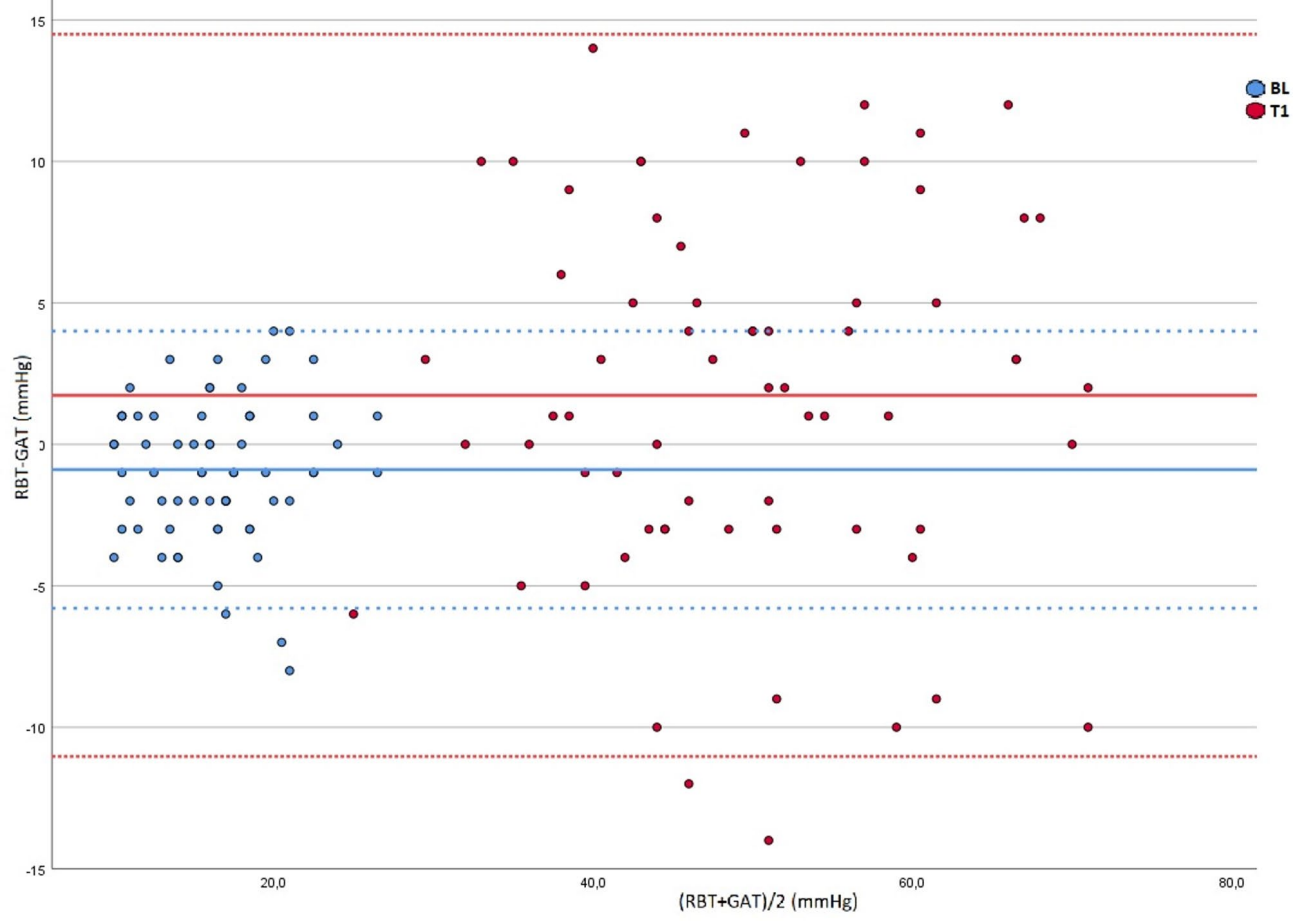
Bland–Altmann scatter plot illustrating the intraocular pressure measurement agreement between rebound tonometry (RBT) and Goldmann applanation tonometry (GAT) at baseline (BL - blue) and at the first post-injection time point (T1 - red). x-axis: (RBT + GAT)/2: the mean RBT and GAT measurements, y axis: RBT − GAT: the difference between the RBT and GAT measurements. The mean measurement difference and limits of 95% agreement are indicated by the corresponding colored continuous bold and dashed lines, respectively.

The agreement between the RBT and GAT measurements was evaluated with a total of 1,206 combined IOP data points and was calculated with the Bland–Altman method. The mean measurement difference at BL was − 0.90 mm Hg between RBT and GAT. The limits of 95% agreement were − 5.79 and 4.01, respectively. Higher IOPs yielded lower levels of agreement. The most pronounced mean measurement difference of 1.73 mm Hg was observed at T1 with limits of 95% agreement at − 11.03 and 14.50 (Fig. 3). As the IOP normalized, the agreement improved again. At D1, the results showed a mean measurement difference of − 0.45 mm Hg with limits of 95% agreement at − 5.70 and 4.81.

Linear mixed-effects model was applied to evaluate IOP changes in phakic (*n* = 12) and pseudophakic (*n* = 55) eyes. BL IOP readings (mean ± SD) for phakic and pseudophakic eyes were 18.00 ± 3.77 and 16.49 ± 4.29 mmHg, respectively. No significant difference was observed at any of the measured time points before or after IVA (*p* = 0.852). Pseudophakic eyes were further investigated. Forty-nine eyes (89.09%) have undergone prior posterior yttrium-aluminum-garnet (YAG) laser capsulotomy; therefore, statistical comparison with patients with an intact capsular bag was not feasible.

Three reflux cases were classified as interventional adverse events. One eye (2 × 2 mm reflux) showed no IOP elevation at T1, whereas the remaining two (both 1 × 1 mm reflux) had a T1 peak of approximately 40 mmHg, with IOP returning to BL levels as described earlier. All eyes with reflux were excluded from the statistical analysis. No adverse events following IVA administration up to postoperative W1 were observed.

## Discussion

This study presents novel prospective information on the detailed acute IOP dynamics following IVA in eyes that have undergone vitrectomy, those filled with SiO, and eyes with unaltered vitreous bodies. The magnitude of the IOP spike and resolution speed of the IOP elevation did not differ significantly among the three groups at any of the measured time points, regardless of the vitreous cavity status. The uniform IOP curves demonstrate that each eye in every cohort received 0.05 mL of additional volume injected intraocularly, resulting in an equal rise in pressure^20^, and a similar settlement phase. Interestingly, the presence of SiO on the trabecular meshwork was not associated with changes in the resolution of post-IVA IOP spikes in this study, even though the prolonged presence of SiO may lead to trabecular inflammation and thus increased outflow resistance^21^. The debate remains on whether eyes that have undergone PPV without SiO implantation are at a higher risk of IOP elevation and/or development of primary open-angle glaucoma^22^. Overall, available data confirm the safety of intra-silicone and post-PPV administration of aflibercept. The status of the vitreous cavity, to the best of our knowledge, should not incite adjustments to IVA treatment protocols regarding volume.

Eyes that had received multiple IVIs and eyes without prior IVI exhibited a similar resolution curve of the IOP spike in the entire population enrolled with the exception of one single timepoint (T5). Potentially altered outflow could be explained by numerous factors. (1) SiO microdroplet contamination of repeated IVIs, and the mostly defined SiO macrodroplet resulting from a single PPV plus SiO tamponade intervention (even with some degree of emulsification) may not behave similarly. The needles and syringes available at the study site were not manufactured without SiO. Although all IVAs were performed using uniform equipment, the potential for additional SiO contamination—and therefore, an additional rise in IOP—cannot be ruled out across the entire cohort. (2) Scleral elasticity is thought to be a further defining element in the normalization of IOP following IVI^18^; however, the literature on this topic is scarce. Healing or scarring of the ports used for 23-G PPV appeared to have lesser effects on the altered scleral biomechanics regarding the degree of meaningful increases in outflow resistance, whereas repeated IVIs may have had an effect. 3) Post-vitrectomy administration of therapeutic TCA can result in late IOP rise^6^, whereas the effect of TCA for intraoperative vitreous visualization on IOP elevation is currently unknown.

Notably, cohorts with consistently shorter AL at < 22.0 mm^23^, or shallower ACD at < 2.4 mm^24^, or those with gonioscopically closed angles may yield different results^25^. In addition, extrapolating these findings to non-Caucasian populations remains uncertain, as well as in patients with impaired trabecular outflow.

Despite a significant age difference between the G-SiO/G-NVIT (*p* = 0.02) and G-SiO/G-PPV groups (*p* = 0.02), the baseline IOP, maximum IOP, and resolution speed of IOP spikes were not affected. This finding aligns with the study reporting the lack of age-related increase in IOP in otherwise healthy individuals^26^. The significantly higher proportion of treatment-naïve eyes to prior IVIs in the G-SiO group before IVA administration were attributed to the indications for IVA being diagnosed after SiO implantations, thus rendering lower pre-enrollment IVI numbers. Balancing these two BL characteristics in later studies could lead to a more precise understanding of the SiO-related IOP mechanisms. The present study cohorts along with indications for IVA reflect real-life scenarios.

The agreement between RBT and GAT was moderate to good at normal or near-normal IOP levels, such as BL and D1, as supported by existing studies^27^. RBT consistently underestimated GAT at all-time points, except for the extremely high values recorded at T1, which showed huge disagreement. Notably, owing to the study design, the two consecutive readings (RBT first followed by GAT) of this hyperacute phase may also display temporal changes along the steep slope of the declining IOP curve. To our knowledge, this is the first study assessing the performance of iCare-100 RBT at excessively high IOP levels, where it appears to be less consistent with GAT. A limitation of this trial is the use of a single GAT measurement, similar to the design employed by Munkwitz et al.^28^. Although multiple readings provide more robust IOP information^29^ and are the recommended methodology in clinical trials^17^, repeated corneal applanation may or may not lead to a significant reduction in IOP^30,31^. Changes in IOP that occur briefly after IVI are not well-mapped, and even with an implanted suprachoroidal sensor, this phase remains largely unexplored^32^. Moreover, the ideal timing for a second GAT measurement is debatable^33^. In our opinion, incorporating additional steps into the algorithm while the patient is undergoing repetitive tonometry would have introduced a novel source of bias.

Lens status had no effect on pre- or post-IVA IOP. In this study, the hypothesis that posterior YAG laser capsulotomy is a risk factor for prolonged IOP elevation^34^ was neither supported nor rejected, as most pseudophakic eyes had already undergone laser capsulotomy before enrollment.

## Conclusions

To our knowledge, this study is the first to publish prospective findings on human cohorts regarding the acute IOP dynamics following the intra-silicone administration of aflibercept, comparing it with vitrectomized eyes that do not have silicone tamponade and eyes with unaltered vitreous bodies. Intra-silicone or post-vitrectomy administration of IVA proved to be safe, with no difference in the level of or resolution of the IOP spike. These eyes are not expected to behave fundamentally differently from eyes with unaltered vitreous in the early postinjection phase. Eyes that had received multiple prior IVIs regardless of the vitreous status exhibited altered normalization of the IOP at a single time point. However, whether this significant difference is clinically relevant, particularly after repeated injections in patients with severe glaucomatous optic nerve damage, remains unknown.

Regarding the monitoring of IOP peak resolution after intra-silicone or post-PPV aflibercept injections, RBT may serve as a fast and convenient alternative for most patients, although it may underestimate IOP. However, it is generally less consistent with GAT at extremely high IOP levels. When precise measurements are required, particularly in patients with concomitant glaucoma, GAT should still be considered the gold standard^35^.

**Table 1:** Indications for intravitreal aflibercept injection. (*DME* diabetic macular edema, *G-NVIT* non-vitrectomized study arm, *G-PPV* Pars plana vitrectomy study arm, *G-SiO* silicone oil study arm, *IGS* Irvine–Gass syndrome, *PDR* proliferative diabetic retinopathy, *RVO* retinal vein occlusion, *wAMD* wet age-related macular degeneration).

	G-SIO (*n* = 22)	G-PPV (*n* = 22)	G-NVIT (*n* = 23)
DME	10	19	17
wAMD	1	3	5
PDR	7	-	-
IGS	4	-	-
RVO	-	-	1

**Table 2:** Baseline characteristics of the three study arms. (*ACD* anterior chamber depth, *AL* axial length, *BL IOP* baseline intraocular pressure, *CCT* central corneal thickness, *F* female, *G-NVIT* non-vitrectomized study arm, *G-PPV* Pars plana vitrectomy study arm, *G-SiO* silicone oil study arm, *ITC* iridotrabecular contact, *IVI* intravitreal injection, *M* male, *S360* 360 degree integrated index based on Shaffer gonioscopic assessment).

	G-SIO (*n* = 22)	G-PPV (*n* = 22)	G-NVIT (*n* = 23)	*P* value
BL IOP (Hgmm)	16.27 ± 3.34	17.68 ± 4.19	16.35 ± 4.96	0.464 ANOVA
AL (mm)	23.41 ± 0.60	23.19 ± 0.74	23.00 ± 1.08	0.263 ANOVA
ACD (mm)	3.72 ± 0.76	3.65 ± 0.84	3.74 ± 0.83	0.932 ANOVA
CCT (um)	543.55 ± 30.76	540.95 ± 46.16	548.30 ± 38.70	0.815 ANOVA
peudophakia (n)	16	21	18	0.122 Pearson Chi-Square test
treatment naïve to prior IVI (n)	17	6	6	< 0.001 Pearson Chi-Square test
S360 (0–16)	14.78 ± 2.17 (16, Q1 = 14, Q3 = 16)	15.23 ± 2.07 (16, Q1 = 15.75, Q3 = 16)	14.46 ± 2.48 (16, Q1 = 12.75, Q3 = 16)	0.265 Kruskal-Wallis
ITC (*n* = 0 degrees)	20	21	19	0.645 Chi square test for independence
age (years)	53.68 ± 18.47	66.59 ± 9.46	65.96 ± 8.73	0.017 Welch ANOVA
sex (M/F)	15/7	13/9	12/11	0.568 Pearson Chi-Square

## Data Availability

The datasets generated and analyzed during the current study are available from the corresponding author on reasonable request.
